# Supplementary material for: Pushing the boundaries of optoacoustic microscopy by total impulse response characterization
Source: Nat Commun. 2020 Jun 9;11:2910. doi: 10.1038/s41467-020-16565-2 (PMC7283257; doi:10.1038/s41467-020-16565-2)
Supplement: Supplementary file 1 — Supplementary Information [file 41467_2020_16565_MOESM1_ESM.pdf]

Pushing the boundaries of optoacoustic microscopy by Total Impulse Response characterization

Seeger et al.

Supplementary Information

Abbreviations

EIR	Electric impulse response
GL	Gold layer
MAP	Maximum amplitude projection
OA	Optoacoustic
OAM	Optoacoustic microscopy
OIR	Optical impulse response
OR	Optical-resolution
PSF	Point spread function
SFR	Spatial frequency response
SIR	Spatial impulse response
SMF	Spatial matched filter
SNR	Signal-to-noise ratio
SOAPs	Spatially-distributed optoacoustic point sources
TIR	Total impulse response

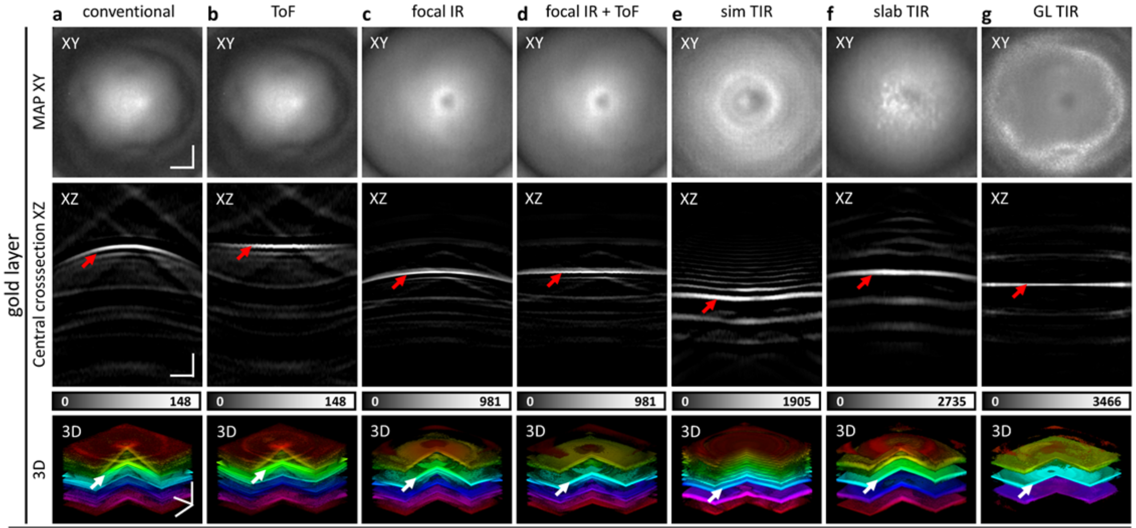

50% legacy					
Sig	23-148 (mean 64.4)	353-981 (mean 548.9)	456-1905 (mean 1042.2)	451-2735 (mean 1232.8)	197-3466 (mean 1188.1)
BG	0-47 (mean 4.3)	0-486 (mean 14.8)	0-1355 (mean 26.4)	0-870 (mean 35.0)	0-762 (mean 23.7)
Mean/mean	14.97 (23.50 dB)	37.09 (31.39 dB)	39.47 (31.93 dB)	35.22 (30.94 dB)	50.13 (34.00 dB)
Max/mean	34.42 (30.73 dB)	66.28 (36.43 dB)	72.16 (37.17 dB)	78.14 (37.86 dB)	146.24 (43.30 dB)

Supplementary Figure 1 **Comparison of different correction methods for optoacoustic microscopy data recorded from a flat gold layer**. The achieved XZ-images are analysed by seeding the main signals corresponding to the gold layer (indicated with the red arrow) with 50 % legacy to segment the main signals from the noise (see table). The conventional data projection using frequency-filtered and Hilbert-transformed signals are depicted as **a** a XY-MAP, a central XZ-cross section and a depth-colour coded 3D rendering. The conventional projection shows a bent surface, signal repetition and artefacts. **b** After applying time-of-flight correction (ToF), the gold layer appears flat in space. **c** Correcting the data with a spatial matched filter (SMF) using the focal impulse response (IR) shows fewer artefacts, a higher SNR and a flatter surface than (a), **d** the latter can be subsequently rectified by ToF correction. **e** Correcting the data with a SMF using a simulated TIR induces various signal repetitions but increases the image SNR and leads to an almost flat surface. **f** Correcting the data with a SMF using the TIR determined with a slab (i.e. axially not a point source) leads to a slightly bent surface, fewer artefacts, and a higher SNR. **g** Correcting the data with a SMF using the TIR determined by the gold layer (GL) achieves the best and flattest depiction, the fewest artefacts and the highest SNR. Scale bar is 100 µm.

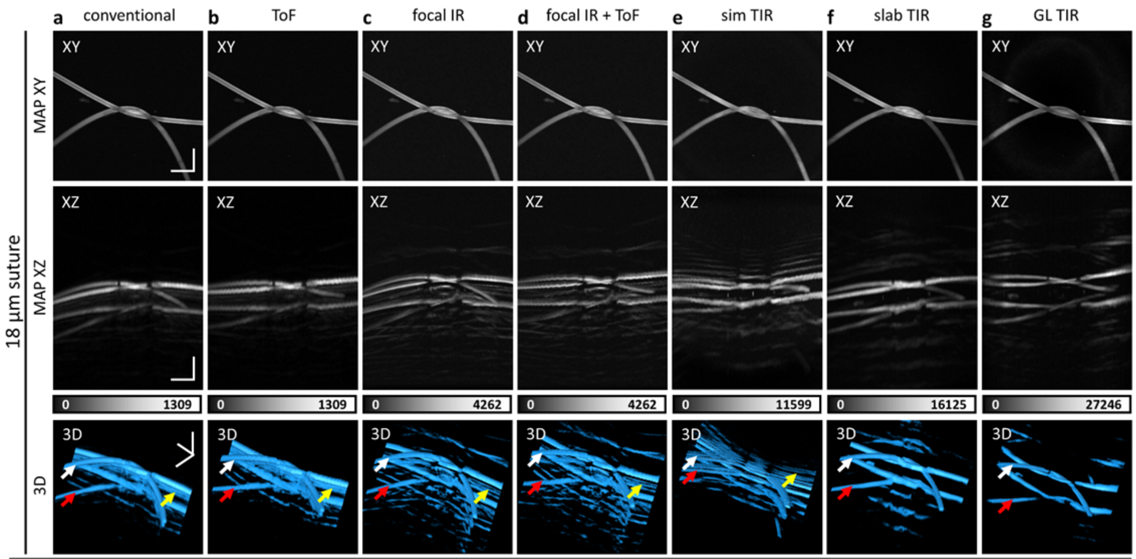

**10% legacy**					
Sig	408-1309 (mean 718.8)	1336-4262 (mean 2277.9)	3371-11599 (mean 6370.2)	3751-16125 (mean 8120.3)	4148-27246 (mean 8748.2)
BG	0-1056 (mean 111.8)	0-3382 (mean 458.9)	0-11902 (mean 1294.5)	0-12023 (mean 1206.2)	0-15449 (mean 1241.1)
Mean/mean	6.43 (16.16 dB)	4.96 (13.91 dB)	4.92 (13.83 dB)	6.73 (16.56 dB)	7.05 (16.96 dB)
Max/mean	11.71 (21.37 dB)	9.28 (19.35)	8.96 (19.04 dB)	13.37 (22.52 dB)	21.95 (26.83 dB)

Supplementary Figure 2 **Comparison of different correction methods for optoacoustic microscopy data recorded from 18 µm black polystyrene sutures**. The achieved XZ-images are analysed by seeding the main signals corresponding to the suture layer (white arrow) with 10 % legacy to segment the main signals from the noise (see table). The conventional data projection using frequency-filtered and Hilbert-transformed signals are depicted as **a** a XY-MAP, a XZ-MAP, and a 3D rendering. The conventional projection shows bent sutures, signal repetitions induced by the ultrasound waves back-reflected from the sample-holding glass substrate (red arrow), and artefacts (yellow arrow). **b** After applying time-of-flight correction (ToF), the sutures appear flat in space. **c** Correcting the data with a spatial matched filter (SMF) using the focal impulse response (IR) shows fewer artefacts, a higher SNR and a flatter suture than (a), **d** the latter can be subsequently rectified by ToF correction. **e** Correcting the data with a SMF using a simulated TIR induces various signal repetitions but increases the image SNR and leads to almost flat sutures. **f** Correcting the data with a SMF using the TIR determined with a slab (i.e. axially not a point source) leads to slightly bent sutures, fewer artefacts, and a higher SNR. **g** Correcting the data with a SMF using the TIR determined by the gold layer (GL) achieves the best and flattest depiction, the fewest artefacts and the highest SNR. Scale bar is 100 µm.

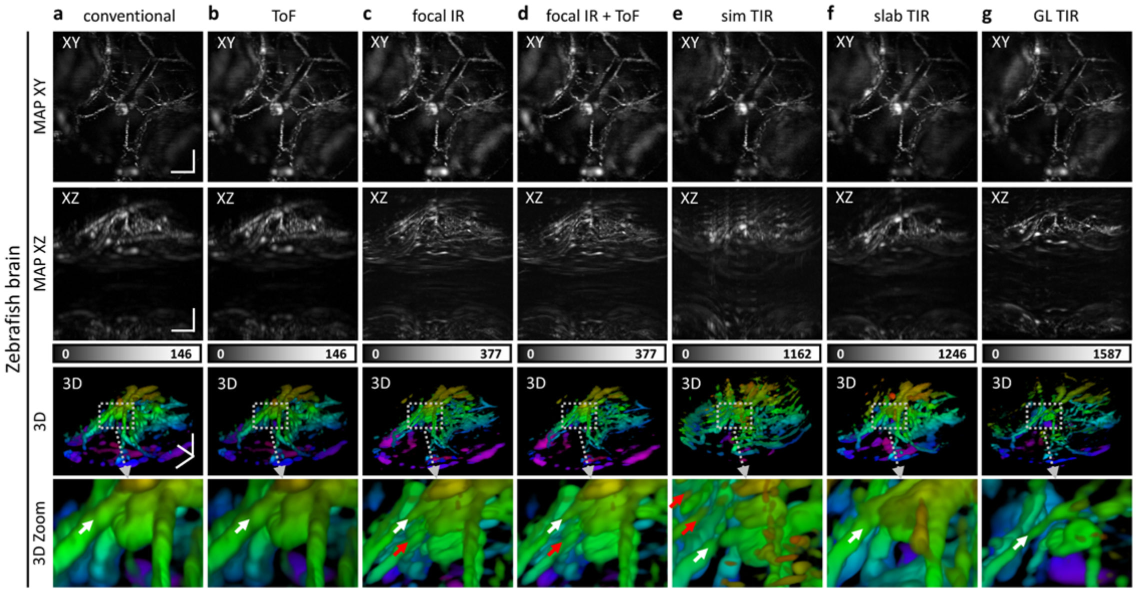

Supplementary Figure 3 **Comparison of different correction methods for optoacoustic microscopy data recorded from *in vivo* zebrafish brain vasculature**. The conventional data projection using frequency-filtered and Hilbert-transformed signals is depicted as **a** a XY-MAP, a XZ-MAP, a depth-colour coded 3D rendering, and a zoomed 3D rendering. The conventional projection shows blurry and axially elongated vessels (white arrow). **b** After applying time-of-flight correction (ToF), the entire brain vasculature appears flatter in space. **c** Correcting the data with a spatial matched filter (SMF) using the focal impulse response (IR) shows fewer artefacts, less signal repetition (red arrow) and a flatter brain than (a), **d** the latter can be subsequently rectified by ToF correction. **e** Correcting the data with a SMF using a simulated TIR induces various signal repetitions (red arrows). **f** Correcting the data with a SMF using the TIR determined with a slab (i.e. axially not a point source) leads to a vanishing of signal repetitions. **g** Correcting the data with a SMF using the TIR determined by the gold layer (GL) achieves the best and flattest depiction, the fewest artefacts, and the sharpest vessels. Scale bar is 100 µm.

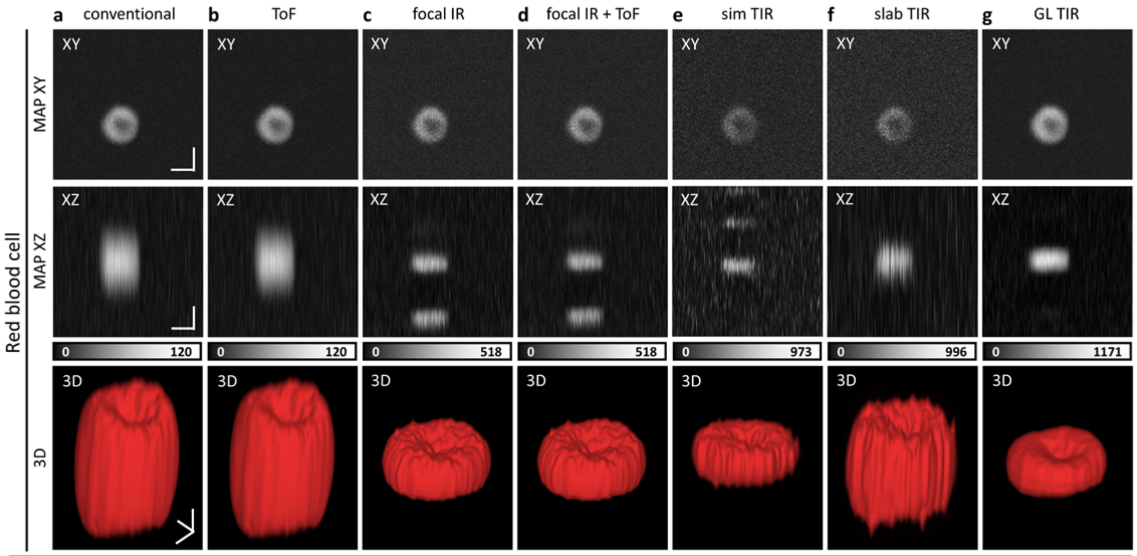

70% legacy					
Sig	60-120 (mean 87.2)	247-518 (mean 341.9)	715-973 (mean 839.7)	838-996 (mean 880.6)	314-1171 (mean 887.0)
BG	0-59 (mean 38.7)	0-458 (mean 204.7)	0-945 (mean 600.3)	0-919 (mean 225.8)	0-347 (mean 168.4)
Mean/mean	2.25 (7.04 dB)	1.67 (4.45 dB)	1.40 (2.92 dB)	3.89 (11.79 dB)	5.26 (14.42 dB)
Max/mean	3.10 (9.83 dB)	2.53 (8.06)	1.62 (4.19 dB)	4.41 (12.89 dB)	6.95 (16.84 dB)

Supplementary Figure 4 **Comparison of different correction methods for optoacoustic microscopy data recorded from a single red blood cell (RBC)**. The conventional data projection using frequency-filtered and Hilbert-transformed signals is depicted as **a** a XY-MAP, a XZ-MAP, and a 3D rendering. The conventional projection shows an elongated RBC. **b** After applying time-of-flight correction (ToF), the RBC seems unchanged due to the small ToF differences across the RBC. **c** Correcting the data with a spatial matched filter (SMF) using the focal impulse response (IR) induces a strong signal repetition and shows a thinner RBC than (a) after windowing out the signal repetitions (c). **d** ToF correction again seems to not affect the images. **e** Correcting the data with a SMF using a simulated TIR induces various signal repetitions. **f** Correcting the data with a SMF using the TIR determined with a slab (i.e. axially not a point source) leads to a blurring of the RBC boundary. **g** Correcting the data with a SMF using the TIR determined by the gold layer (GL) achieves the best and flattest depiction, the fewest artefacts, and the sharpest RBC. Furthermore, an increase in SNR leads to a clearer boundary and, thus, to a smoother rendering. Scale bar is 5 µm.

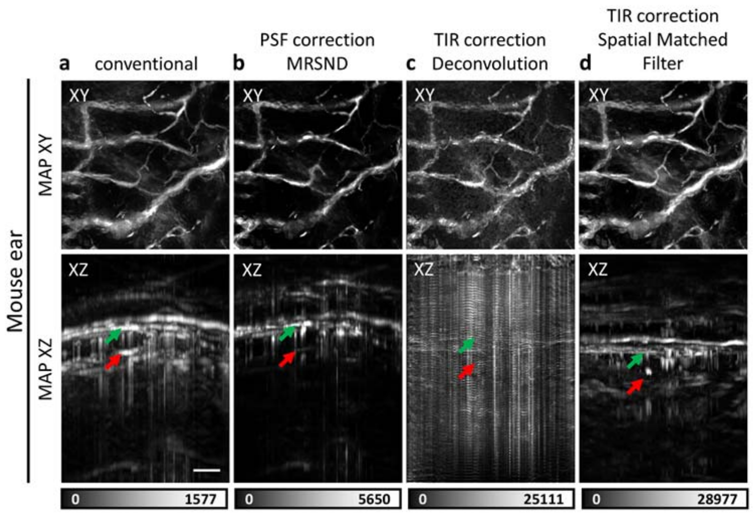

Supplementary Figure 5 **Comparison of different correction methods for optoacoustic microscopy data recorded from *in vivo* mouse ear vasculature**. The conventional data projection using frequency-filtered and Hilbert-transformed signals is depicted as **a** a XY-MAP, and a XZ-MAP. The conventional projection shows strong signal repetitions (red arrow) and a curved vasculature (green arrow). **b** Correcting the projected data with a spatially depended PSF determined with 1 µm microspheres at 10 lateral offset positions shows less artefacts, a flatter depiction, and a higher SNR. **c** Correcting the data using the TIR determined from the gold layer in form a deconvolution at the signal level induces strong artefacts but a flat and sharp shadow of the main signal (green arrow) without signal repetition. **d** Correcting the data with a SMF using the TIR determined by the gold layer (GL) at the signal level achieves the best and flattest depiction, fewer artefacts than (b), and a vessel sharpness comparable to (c). Scale bar is 100 µm.

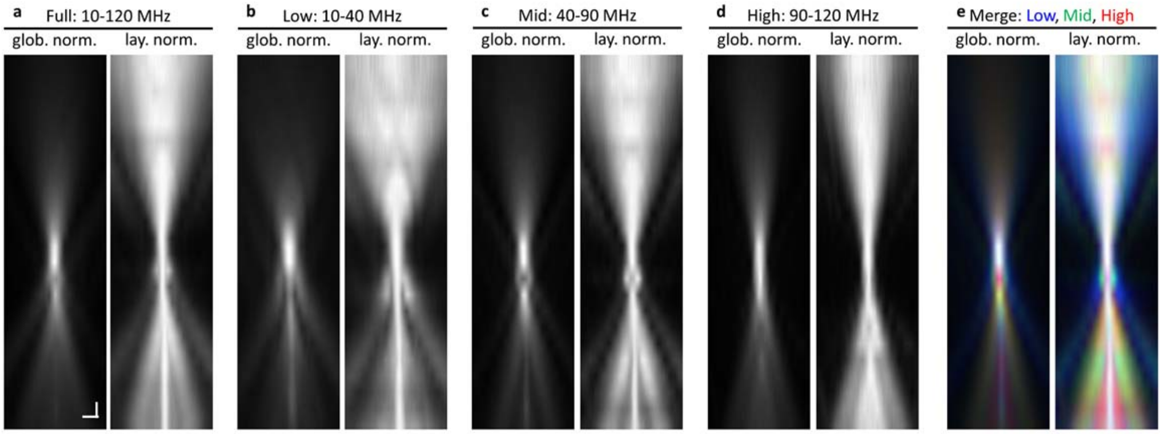

Supplementary Figure 6 **Central plane of the transducer's sensitivity field** for different frequency ranges in a globally normalized or a per-axial-layer normalized depiction. **a** The depiction generated using the entire -6 dB bandwidth of the transducer (i.e. 10-120 MHz) reveals an asymmetric sensitivity field with a bulge-like structure and side lobes. **b** The depiction using a low frequency band (i.e. 10-40 MHz) shows strong side lobes and no bulge. **c** The depiction using a middle frequency band (i.e. 40-90 MHz) shows a strong bulge and intermediate dominant side lobes. **d** The depiction using a high frequency band (i.e. 90-120 MHz) shows neither side lobes nor the bulge, but a sharp acoustic focal zone. **e** The RGB-overlay of the three separately generated depictions confirm the findings that the side lobes are predominantly produced by low frequencies, the bulge structure mainly by middle frequencies, and the focal region predominantly by high frequencies. Scale bar is 100 µm.

39

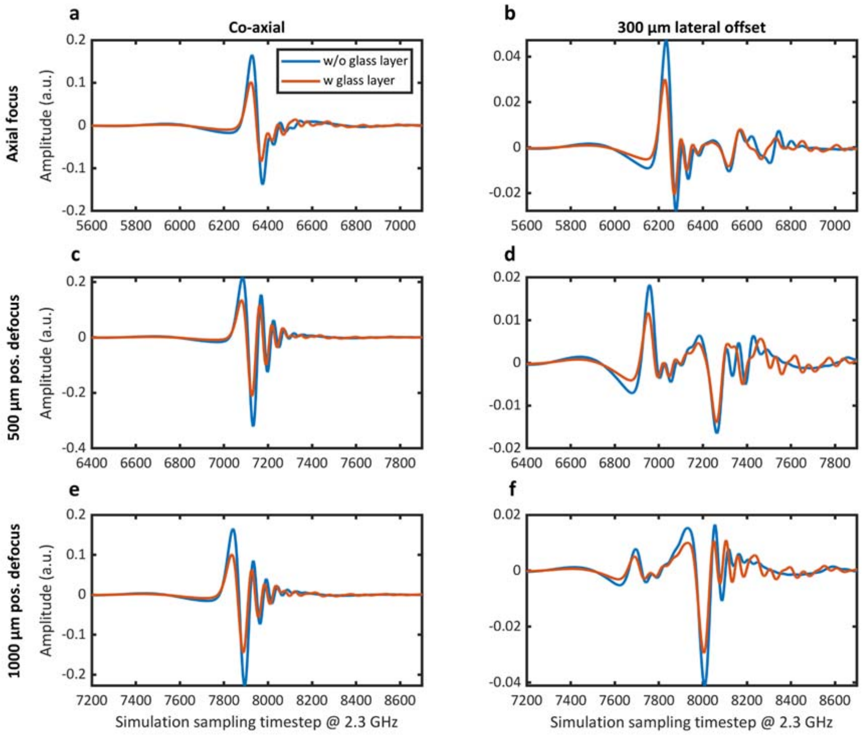

Supplementary Figure 7 **Influence of the glass layer on the simulated TIR** for different positions within the simulated volume performed analogous to Fig. 4. **a** The 3D simulation with and without a glass layer beneath the simulated acoustic point source lead to a negligible difference in the recorded signal by the simulated transducer in the acoustic focus. Similar findings are observed for **b** a 300 µm lateral offset within the axial focal plane, **c-d** at 500 µm positive defocus, and **e-f** at 1000 µm positive defocus.

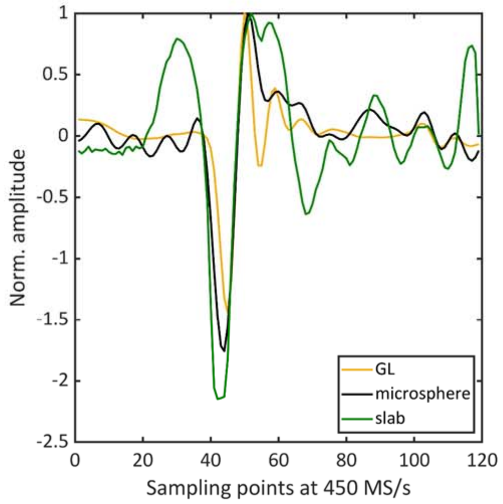

Supplementary Figure 8 **Comparison of signals recorded from the gold layer (GL), a 1 µm microsphere, and a slab** as possible specimens to generate point sources. The similarity of the signals was assessed using the relative squared L^2-error, which yielded a relative error of 0.28 between the GL and the microsphere and 0.90 between the slab and the microsphere.

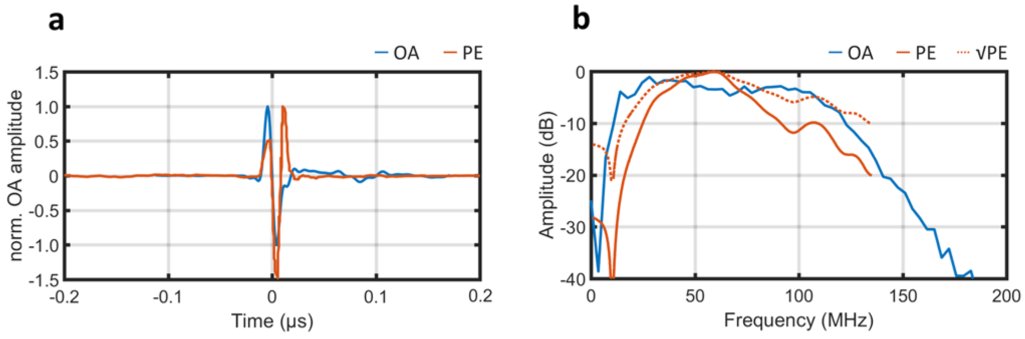

Supplementary Figure 9 **Comparison of ultrasound transducer characterized by optoacoustic and ultrasound pulse-echo. a** Raw signals received from the acoustic focus by the optoacoustic approach using the GNL and pulse-echo ultrasound extracted from the manufacturer datasheet on a centered time-axis. The PE technique comprised a third peak not being visible in the OA technique. **b** Corresponding frequency content of both characterization techniques. OA characterization led to a -10 dB bandwidth of ~10 MHz to ~120 MHz, PE characterization to ~25 MHz to ~90 MHz, reflecting both the EIR of the transducer acting twice on the ultrasound wave and the ultrasound wave passes twice through the focal length in PE characterization. The square root of the PE-sensed frequencies approximate the OA bandwidth better. Abbreviations: OA, optoacoustic; PE, pulse-echo, $\sqrt{PE}$, square root of PE frequency content.

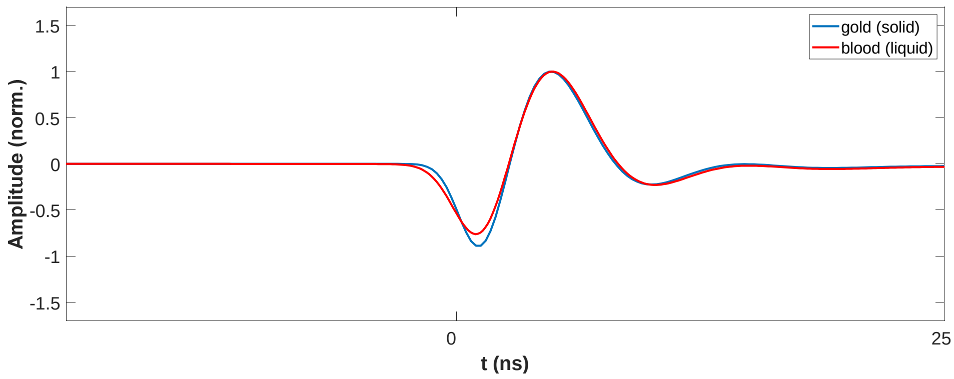

Supplementary Figure 10 **Comparison of simulated 1 μm spherical optoacoustic sources.** Simulated signals generated from a solid (gold) or liquid (blood) spheres.
