# Supplementary material for: Pushing the boundaries of optoacoustic microscopy by total impulse response characterization
Source: Nat Commun. 2020 Jun 9;11:2910. doi: 10.1038/s41467-020-16565-2 (PMC7283257; doi:10.1038/s41467-020-16565-2)
Supplement: Supplementary file 3 — Description of Additional Supplementary Files [file 41467_2020_16565_MOESM3_ESM.pdf]

Description of Additional Supplementary Files

Supplementary Movie 1: Comparison between simulation and experiment of TIR characterization

Supplementary Movie 2: Image improvement through TIR correction of a mouse ear vasculature

Supplementary Movie 3: Image improvement through TIR correction of a mouse ear microcapillary

Supplementary Movie 4: Image improvement through TIR correction of a human red blood cell

Supplementary Movie 5: Image improvement through TIR correction of a zebrafish brain vasculature
